# Behaviors Associated With Improved Relationship Satisfaction: Examining Change in the Relationship Checkup

**DOI:** 10.1111/famp.70162

**Published:** 2026-06-20

**Authors:** Erica A. Mitchell, Patricia N. E. Roberson, Desirée S. Woodle, James V. Cordova, Kristina Coop Gordon

**Affiliations:** ^1^ Department of Human Development and Family Studies Michigan State University Lansing Michigan USA; ^2^ College of Nursing University of Tennessee Knoxville Tennessee USA; ^3^ Department of Psychology University of Tennessee Knoxville Tennessee USA; ^4^ Department of Psychology Clark University Worcester Massachusetts USA; ^5^ College of Education, Health, and Human Sciences University of Tennessee Knoxville Tennessee USA

**Keywords:** brief intervention, couples, relationship satisfaction

## Abstract

Relationship distress and dissolution are reported by about one‐third of couples in the United States. Despite evidence that couple therapy is an effective treatment for relationship distress, many couples encounter barriers (e.g., cost, time, transportation, childcare) to accessing this kind of relationship help. The Relationship Checkup is a two‐session, individualized intervention where each couple receives evidence‐based suggestions to address specific relationship concerns. The Relationship Checkup was delivered as a home visitation intervention in the Southeastern region of the United States. The current study (*N* = 1298 individuals; *N* = 649 couples) uses Dyadic Latent Growth Curves models to examine associations between short‐term (i.e., pre‐intervention to 1‐month post‐intervention) change in an indicator of relationship quality (i.e., constructive communication, intimate safety, and relationship aggression) and longer‐term change (i.e., pre‐intervention to 6‐months post‐intervention) in relationship satisfaction. Specifically, we examine how change in one's own indicator of relationship quality influences changes in one's own and one's partner's relationship satisfaction. Across all indicators of relationship quality, short‐term change for Partner A (i.e., the partner who requested the Relationship Checkup) is associated with longer‐term change in satisfaction for self and partner. For Partner B, short‐term change in each indicator is associated with longer‐term change in satisfaction for self, but not for partner. Supplemental analyses indicate improvements are generally maintained for relationship aggression, while improvements for intimate safety and constructive communication generally decelerate following the intervention. Attuning to the identified areas of concern for the partner requesting the Relationship Checkup may be an effective approach to practice.

## 1 Introduction

In the United States, about 32% of married couples report experiencing relationship distress (Whisman et al. 2008), and about 39% of marriages end in divorce (Centers for Disease Control 2023). Thus, it is evident that relationship distress is common. Relationship distress is associated with increased risk for mental health disorders (e.g., depression, anxiety; Whisman 2007); these associations are found cross‐culturally (McShall and Johnson 2015). Relationship distress is also a risk factor for physical health problems (e.g., cardiovascular disease) and higher mortality rates (Robles et al. 2014). The negative impact of relationship distress extends beyond the individual; for example, relationship distress is associated with more adjustment problems for children (Harold et al. 2004). The outcomes associated with relationship distress are concerning, and there is a clear need for effective interventions to develop and maintain healthy, stable relationships. Despite couple therapy being an effective intervention for relationship distress (e.g., Roddy et al. 2020) many couples encounter several barriers to accessing this type of relationship help (e.g., money, time, transportation, childcare). Thus, there needs to be interventions that are not only effective but are accessible to couples in greatest need.

The Relationship Checkup is a two‐session assessment and feedback intervention designed to be the relationship health equivalent of an annual physical health checkup. In this way the Relationship Checkup can provide preventative care to couples before they become distressed. The Relationship Checkup is rooted in motivational interviewing (Miller and Rollnick 2002) and Integrative Behavioral Couple Therapy (IBCT; Christensen and Jacobson 1991; Cordova et al. 1998). It aims to activate couples to take care of their relationship while fostering greater acceptance and deeper intimacy. Expression of vulnerable emotions is seen as a pathway to achieve deeper intimacy, and a way to identify softer emotions and understandable reasons behind each partners' behaviors to promote greater acceptance in the relationship (see Cordova et al. 2005, 2014 for more information). The Relationship Checkup is an individualized intervention—it is tailored to each couple's unique needs and has been adapted across diverse populations (e.g., Gray et al. 2022). As in all couple treatment, one partner typically initiates services on behalf of the relationship. This can be an indicator of motivation to actively participate in treatment, or in some cases of level of distress (e.g., Hodge et al. 2024). It is important for interventionists to be aware of each partner's level of motivation at the start of treatment, as a strong alliance with both partners is associated with better treatment outcomes (e.g., Anker et al. 2010). This may be particularly critical for a very brief intervention like the Relationship Checkup where rapport building and relationship change are quickly occurring.

Given this is a systemic intervention, it is also important to account for nonindependence as changes in relationship dynamics are occurring during and following the intervention (e.g., Oka and Whiting 2013). For example, one partner's experience of changes in communication during the intervention may impact both their own and their partner's sense of satisfaction in the relationship. In an effort to better understand the associations between short‐term change in relationship quality and longer‐term change in relationship satisfaction, it is imperative to closely examine how short‐term changes for each partner influence longer‐term changes for oneself and one's partner. A careful examination of the data in this way has practical implications for understanding how this intervention influences relationship change over time, thus informing more effective treatment and long‐term, sustainable relationship change.

Prior studies with the Relationship Checkup have found improvements in acceptance, intimate safety, and relationship satisfaction (Cordova et al. 2014). Additionally, improvements in acceptance are associated with early (i.e., 2‐weeks post‐intervention) and long‐term (i.e., 1‐year post‐intervention) improvements in relationship satisfaction, and early improvements in intimate safety are marginally associated with long‐term (i.e., 1‐year post‐intervention) improvements in satisfaction (Hawrilenko et al. 2016). Furthermore, low marital quality is associated with a greater likelihood of seeking out the Relationship Checkup. More specifically, wives' help seeking is significantly impacted by their own perception of marital quality, and husbands' help seeking is significantly impacted by their wives' perceptions of marital quality more than their own (Fleming and Cordova 2012).

Gordon et al. (2019) modified the Relationship Checkup (a) to be more inclusive of relationship status (i.e., cohabitating and married couples) and (b) to allow participants to choose to receive the intervention in their home or at a local clinic. Most participants chose to receive the Relationship Checkup in their home, which likely helped to reduce common access barriers (e.g., transportation, time). This study oversampled couples with low‐income and economic marginalization. Participants reported being satisfied with the Relationship Checkup, and relationship satisfaction significantly improved from baseline to 6‐month follow‐up. Additionally, significant improvements in communication, psychological and physical aggression, and intimate safety from baseline to 1‐month follow‐up were reported. Further, moderation analyses suggested that distressed participants experienced significantly larger improvements in these indicators of relationship quality.

### 1.1 Current Study

Using data from Gordon et al. (2019) study, the current study aims to understand the associations between short‐term change in indicators of relationship quality (i.e., communication, intimate safety, and relationship aggression) and longer‐term change in relationship satisfaction. We will explore these associations in piece meal, e.g., from pre‐intervention through 1‐month post intervention and from 1‐month post intervention through 6‐months post intervention. This study extends current research on the Relationship Checkup by examining these associations with a community‐based sample with a wider range of income and education levels. Based on findings from prior research with this sample, we propose the following hypotheses:

Hypothesis 1a *Changes in an individual's constructive communication from pre‐intervention to* 1*‐month post‐intervention will be linked to a change in their own relationship satisfaction from pre‐intervention through* 6*‐months post‐intervention*.

Post Hoc Hypothesis 1a i. Changes in an individual's constructive communication from pre‐intervention to 1‐month post‐intervention will be linked to changes in *their own* relationship satisfaction from pre‐intervention through 1‐month post‐intervention.

Post Hoc Hypothesis 1a ii: Changes in an individual's constructiveness communication from pre‐intervention to 1‐month post‐intervention will be linked to changes in *their own* relationship satisfaction from 1‐month post‐intervention through 6‐months post‐intervention.

Hypothesis 1b *Changes in an individual's constructive communication from pre‐intervention to* 1*‐month post‐intervention will be linked to a change in their partner's relationship satisfaction from pre‐intervention through* 6*‐months post‐intervention*.

Post Hoc Hypothesis 1b i. Changes in an individual's constructive communication from pre‐intervention to 1‐month post‐intervention will be linked to change in *their partner's* relationship satisfaction from pre‐intervention through 1‐month post‐intervention.

Post Hoc Hypothesis 1b ii. Changes in an individual's constructive communication from pre‐intervention to 1‐month post‐intervention will be linked to change in *their partner's* relationship satisfaction from 1‐month post‐intervention through 6‐months post‐intervention.

Hypothesis 2a *Changes in an individual's intimate safety from pre‐intervention to* 1*‐month post‐intervention will be linked to a change in their own relationship satisfaction from pre‐intervention through* 6*‐months post‐intervention*.

Post Hoc Hypothesis 2a i: Changes in an individual's intimate safety from pre‐intervention to 1‐month post‐intervention will be linked to change in *their own* relationship satisfaction from pre‐intervention through 1‐month post‐intervention.

Post Hoc Hypothesis 2a ii: Changes in an individual's intimate safety from pre‐intervention to 1‐month post‐intervention will be linked to changes in *their own* relationship satisfaction from 1‐month post‐intervention through 6‐months post‐intervention.

Hypothesis 2b *Changes in an individual's intimate safety from pre‐intervention to* 1*‐month post‐intervention will be linked to a change in their partner's relationship satisfaction from pre‐intervention through* 6*‐months post‐intervention*.

Post Hoc Hypothesis 2b i: Changes in an individual's intimate safety from pre‐intervention to 1‐month post‐intervention will be linked to changes in *their partner's* relationship satisfaction from pre‐intervention through 1‐month post‐intervention.

Post Hoc Hypothesis 2b ii: Changes in an individual's intimate safety from pre‐intervention to 1‐month post‐intervention will be linked to changes in *their partner's* relationship satisfaction from 1‐month post‐intervention through 6‐months post‐intervention.

Hypothesis 3a *Changes in an individual's relational aggression from pre‐intervention to* 1*‐month post‐intervention will be linked to a change in their own relationship satisfaction from pre‐intervention through* 6*‐months post‐intervention*.

Post Hoc Hypothesis 3a i: Changes in an individual's relational aggression from pre‐intervention to 1‐month post‐intervention will be linked to changes in *their own* relationship satisfaction from pre‐intervention through 1‐month post‐intervention.

Post Hoc Hypothesis 3a ii: Changes in an individual's relational aggression from pre‐intervention to 1‐month post‐intervention will be linked to changes in *their own* relationship satisfaction from 1‐month post‐intervention through 6‐months post‐intervention.

Hypothesis 3b *Changes in an individual's relational aggression from pre‐intervention to* 1*‐month post‐intervention will be linked to a change in their partner's relationship satisfaction from pre‐intervention through* 6*‐months post‐intervention*.

Post Hoc Hypothesis 3b i: Changes in an individual's relational aggression from pre‐intervention to 1‐month post‐intervention will be linked to changes in *their partner's* relationship satisfaction from pre‐intervention through 1‐month post‐intervention.

Post Hoc Hypothesis 3b ii: Changes in an individual's relational aggression from pre‐intervention to 1‐month post‐intervention will be linked to changes in *their partner's* relationship satisfaction from 1‐month post‐intervention through 6‐months post‐intervention.

## 2 Method

### 2.1 Procedures

This research was approved by the Institutional Review Board at the University of Tennessee, Knoxville. We report how we determined our sample size, all data exclusions (if any), all manipulations, and all measures in the study. All participants were at least 18 years old and self‐identified as being in a committed intimate relationship (cohabitating or married). For purposes of the current study, “Partner A” refers to the partner who scheduled the Relationship Checkup intervention. All participants completed an informed consent and a baseline questionnaire packet, received the Relationship Checkup assessment and feedback sessions with a trained facilitator, and completed a follow‐up questionnaire packet approximately one month and six months after the feedback session. The 6‐month follow‐up was a reduced questionnaire packet that only included relationship satisfaction to improve participant retention. Participants were compensated up to $150 for participation in the study (see Gordon et al. 2019 for a detailed explanation of the study procedures).

### 2.2 Participants

Of the enrolled participants (*N* = 1298 individuals; *N* = 649 couples), 59% of couples were married and 41% were cohabitating; 95% were different‐sex couples; 58% had at least one child under the age of 18 living in the home. Racially, 80% of participants identified as White, 15% identified as Black, and < 5% identified as either Pacific Islander, Native American, or Asian. In terms of ethnicity, 8% identified as Hispanic. This appears to appropriately reflect the Appalachian region where participants were sampled (the county 2010 census indicates that the population was 86% White, 9% Black, and 2% Hispanic). Participants in the age range of 25–34 made up the largest age group (36%), followed by 35–44 (36%), 45–54 (16%), 18–24 (13%), and 55 and older (8%). For women, 30% worked full‐time, 28% were unemployed, 18% worked part‐time, 13% were disabled, 8% were students, and 2% were retired. Also, for women, 49% had earned a high school diploma/GED or less, and 66% earned less than $20,000 annually. For men, 53% reported working full‐time, 17% were unemployed, 12% worked part‐time, 12% were disabled, 3% were retired, and 2% were students. Also, for men, 53% had earned a high school diploma/GED or less, and 50% earned less than $20,000 annually. When accounting for the number of children living in the home, 27% of couples lived at or under the poverty line. Poverty was computed based on the total household income (sum of the two individual partners' self‐reported income), the number of individuals (children and both romantic partners) that income supports, and the 2016 poverty threshold (0 = above the poverty line, 1 = at/below the poverty line). A majority (89%) of couples completed both intervention sessions.

### 2.3 Measures

#### 2.3.1 Relationship Satisfaction

Relationship satisfaction was measured with the Couple Satisfaction Index (CSI‐16; Funk and Rogge 2007), a 16‐item self‐report scale measuring general satisfaction in the relationship. Total scores range from 0 to 81 with higher scores indicating greater satisfaction. Typical items include, “My relationship with my partner makes me happy” and “How well does your partner meet your needs?” The CSI‐16 is highly correlated with other measures of satisfaction (*r =* 0.89–0.96), and can appropriately distinguish between non‐distressed and distressed relationships (Funk and Rogge 2007). Using the recommended cutoff score of 51.5 (Funk and Rogge 2007), approximately 32% of participants reported relationship distress at baseline. In this study, CSI demonstrated good internal consistency (baseline *α* = 0.97; 1‐month follow‐up *α* = 0.97; 6‐month follow‐up *α* = 0.97).

#### 2.3.2 Constructive Communication

Constructive communication was measured with the Communication Patterns Questionnaire‐Short Form (CPQ‐SF; Heavey et al. 1996), an 11‐item self‐report questionnaire which measures communication patterns used during conflict. The scale is made up of four subscales, with higher scores on each subscale indicating a greater likelihood of using that communication pattern. Total scores range from 0 to 88, and negative communication items were reverse scored so that higher total scores indicate healthier communication patterns. Typical items include, “When some problem arises in the relationship, both you and your partner try to discuss the problem” and “During a discussion of a relationship problem, both you and your partner express your feelings to each other.” In this study, CPQ‐SF demonstrated good internal consistency (baseline *α* = 0.83; 1‐month follow‐up *α* = 0.84).

#### 2.3.3 Intimate Safety

Intimate safety was measured with the Intimate Safety Questionnaire‐Short Form (ISQ‐SF; Cordova and Scott 2001; Gordon et al. 2019), a 10‐item questionnaire measuring the level of vulnerability in the relationship. Total scores range from 0 to 40 with higher scores indicating greater safety. Typical items include, “I feel comfortable telling my partner things I would not tell anyone else” and “I enjoy sharing my successes with my partner.” In the current study, the ISQ‐SF demonstrated good internal consistency (baseline: *α* = 0.89; 1‐month follow‐up: *α* = 0.86).

#### 2.3.4 Relationship Aggression

Relationship aggression was measured with the Conflict Tactics Scale (CTSS; Straus 1979), a 13‐item self‐report measure of intimate partner conflict and violence. For each item, participants indicated if the behavior ever occurred (1 = *yes*, 2 = *no*, 3 = *don't know*) and if the behavior occurred during the past year (0 = *never* to 5 = *more than once a month*). Total scores range from 0 to 44 with higher scores indicating more relationship aggression. Typical items include, “Argued heatedly but short of yelling” and “Threw something, but not at my partner, or smashed something.” In this study, the CTS demonstrated acceptable internal consistency (baseline: *α* = 0.80; 1‐month follow‐up: *α* = 0.75). See Table 1 for correlations among all variables across time points.

**TABLE 1:** Correlations among all variables pre‐ and post‐intervention.

Variable.	(1)	(2)	(3)	(4)	(5)	(6)	(7)	(8)	(9)	(10)	(11)	(12)	(13)	(14)	(15)	(16)	(17)	(18)
Relationship Satisfaction A—Pre (1)	56.64 (18.60)																	
Relationship Satisfaction B—Pre (2)	0.70**	59.95 (16.61)																
Relationship Satisfaction A – 1 month (3)	0.75**	0.54**	62.63 (17.96)															
Relationship Satisfaction B—1 month (4)	0.55**	0.68**	0.64**	65.31 (13.93)														
Relationship Satisfaction A – 6 month (5)	0.66**	0.55**	0.67**	0.53**	62.07 (17.80)													
Relationship Satisfaction B – 6 month (6)	0.46**	0.58**	48**	0.70**	0.63**	65.26 (14.52)												
Constructive Communication A—Pre (7)	0.64**	0.53**	0.51**	0.39**	0.42**	0.36**	53.32 (17.05)											
Constructive Communication B‐ Pre (8)	0.48**	0.62**	0.41**	0.39**	0.30**	0.38**	0.58**	56.24 (17.10)										
Constructive Communication A – 1 month (9)	0.56**	0.42**	0.66**	0.47**	0.52**	0.34**	0.64**	0.46**	61.20 (17.08)									
Constructive Communication B – 1 month (10)	0.40**	0.48**	0.46**	0.59**	0.41**	0.46**	0.46**	0.63**	0.59**	61.91 (16.33)								
Intimate Safety A—Pre (11)	0.72**	0.48**	0.61**	0.45**	0.54**	0.41**	0.59**	0.42**	0.52**	0.38**	2.82 (0.84)							
Intimate Safety B—Pre (12)	0.49**	0.49**	0.40**	0.53**	0.38**	0.40**	0.46**	0.56**	0.36**	0.46**	0.47**	2.84 (0.79)						
Intimate Safety A—1 month (13)	0.56**	0.41**	0.73**	0.46**	0.58**	0.38**	0.45**	0.33**	0.60**	0.39**	0.72**	0.34**	3.07 (0.76)					
Intimate Safety B—1 month (14)	0.40**	0.53**	0.50**	0.68**	0.42**	0.51**	0.34**	0.43**	0.39**	0.56**	0.36**	0.64**	0.38**	3.02 (0.72)				
Relational Aggression A‐ Pre (15)	−0.28**	−0.26**	−0.19**	−0.13*	−0.14*	−0.22**	−0.44**	−0.38**	−0.29**	−0.25**	−0.20**	−0.24**	−0.13**	−0.15**	26.56 (9.64)			
Relational Aggression B‐ Pre (16)	−0.30**	−0.38**	−0.23**	−0.24**	−0.12*	−0.19**	−0.36**	−0.49**	−0.27**	−0.29**	−0.19**	−0.30**	−0.11*	−0.17**	0.43**	25.42 (9.22)		
Relational Aggression A—Post (17)	−0.23**	−0.16*	−0.24**	−0.18**	−0.15*	−0.23**	−0.31**	−0.29**	−0.38**	−0.32**	−0.24**	−0.17**	−0.20**	−0.15**	0.58**	0.24**	23.25 (8.90)	
Relational Aggression B—Post (18)	−0.15**	−0.18*	−0.16**	−0.15**	−0.12	−0.18**	−0.25**	−0.38**	−0.31**	−0.38**	−0.18**	−0.23**	0.10	−0.16**	0.37**	0.55**	0.46**	23.32 (9.57)

*Note:* Means (standard deviation) are in the diagonal. *N* = 1298.

* *p* < 0.05.

** *p* < 0.001.

### 2.4 Analytic Strategy

**FIGURE 1:**
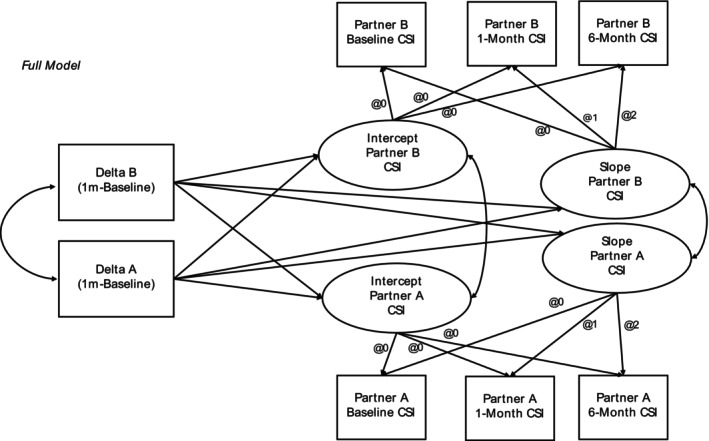
Delta scores were calculated to assess change in each independent variable (i.e., constructive communication, intimate safety, and relational aggression). The slope and intercept of the outcome variable (i.e., relationship satisfaction) across the three time points (i.e., baseline, 1‐month follow‐up, and 6‐month follow‐up) for partner A (i.e., the partner who signed up for the Relationship Checkup intervention) and partner B were regressed onto the delta scores of their own independent variable and their partner's.

**FIGURE 2:**
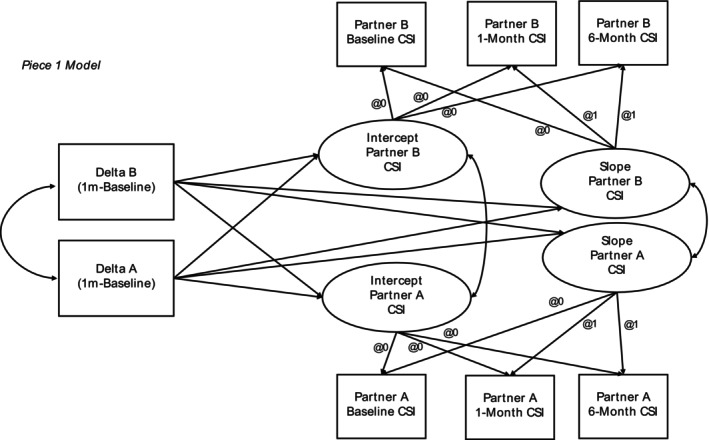
The trajectory was further examined using two sets of partial models. This figure represents the first partial model using baseline and 1‐month follow‐up time points.

**FIGURE 3:**
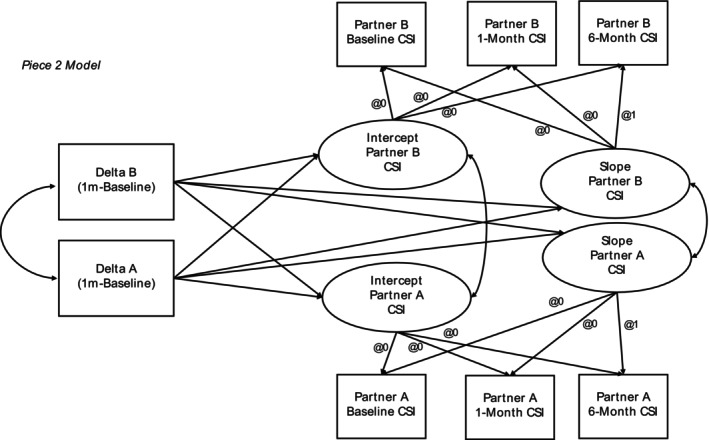
The trajectory was further examined using two sets of partial models. This figure represents the second partial model using 1‐month follow‐up and 6‐month follow‐up time points.

To test all hypotheses, we used Dyadic Latent Growth Curve models where dependent partner observations are correlated to account for the dependence of the data (Duncan et al. 2013; Planalp et al. 2016; Wickrama et al. 2021). Specifically, we ran three separate dyadic models for each independent variable (i.e., constructive communication, intimate safety, and relational aggression). This method is consistently used with longitudinal dyadic data (e.g., Gordon et al. 2019; Roberson et al. 2024). We calculated delta scores to assess change in the independent variables (e.g., relational aggression 1‐month post‐intervention—relational aggression pre‐intervention = delta relational aggression). The slope and intercept of the outcome variable (i.e., relationship satisfaction) across the three time points (i.e., baseline, 1‐month follow‐up, and 6‐month follow‐up) for partner A (i.e., the partner who signed up for the Relationship Checkup intervention) and partner B were regressed onto the delta scores of their own independent variable and their partner's (see Figure 1). Additionally, to examine pieces of the trajectory following the intervention across the three times we re‐ran two sets of partial models: (a) piece 1—baseline and 1‐month follow‐up (see Figure 2) and (b) piece 2–1‐month follow‐up and 6‐month follow‐up (see Figure 3). Effect sizes (i.e., Cohen's d) were used to compare partial model results and full model results (see Table 2). Further, we conducted *Χ* ^*2*^ difference tests to determine if actor and partner parameter estimations were significantly different from one another; a significant *Χ* ^*2*^ (*p* < 0.05) indicates that the parameters are not statistically equal.

**TABLE 2:** Results of dyadic latent growth curve models for change in couple satisfaction.

Model.	Intercept CSI A		Slope CSI A			Intercept CSI B		Slope CSI B		
	B (SE)	Cohen's d	B (SE)	Cohen's d	*Χ* ^*2*^ diff^a^	B (SE)	Cohen's d	B(SE)	Cohen's d	*Χ* ^*2*^ diff^b^
Model 1: Constructive communication										
Baseline to 6‐month (full)										
Delta Partner A	−0.04 (0.04)	−0.04	0.14 (0.03)**	0.18	*p* = 0.90	−0.03 (0.03)	−0.04	0.02 (0.03)	0.03	*p* < 0.001
Delta Partner B	−0.01 (0.04)	−0.01	0.12 (0.03)**	0.16		−0.04 (0.03)	−0.05	0.17 (0.03)*	0.22	
Actor Effect Slope *Χ* ^*2*^ diff^c^	*p* = 0.45									
Partner Effect Slope *Χ* ^*2*^ diff^d^	*p* = 0.02									
Baseline to 1‐month (piece 1)										
Delta Partner A	—	—	0.28 (0.04)**	0.27	*p* = 0.05	—	—	0.05 (0.04)	0.05	*p* < 0.001
Delta Partner B	—	—	0.14 (0.04)*	0.14		—	—	0.32 (0.04)**	0.31	
1‐ to 6‐month (piece 2)										
Delta partner A	—	—	0.07 (0.07)	0.04	*p* = 0.14	—	—	−0.04 (0.06)	−0.03	*p* = 0.03
Delta partner B	—	—	0.23 (0.06)**	0.15		—	—	0.15 (0.06)*	0.09	
Model 2: intimate safety										
Baseline to 6‐month (full)										
Delta partner A	−0.31 (0.83)	−0.01	3.82 (0.68)**	0.22	*p* = 0.31	−0.26 (0.79)	−0.01	0.55 (0.59)	0.04	*p* < 0.001
Delta partner B	−0.54 (0.83)	−0.02	2.73 (0.70)**	0.15		−1.46 (0.76)*	−0.08	4.21 (0.64)**	0.26	
Actor effect slope *Χ* ^*2*^ diff^c^	*p* = 0.66									
Partner effect slope *Χ* ^*2*^ diff^d^	*p* = 0.03									
Baseline to 1‐month (piece 1)										
Delta partner A	—	—	7.19 (0.87)*	0.32	*p* = 0.10	—	—	1.47 (0.88)*	0.06	*p* < 0.001
Delta partner B	—	—	4.97 (0.90)*	0.28		—	—	7.60 (0.84)*	0.35	
1‐ to 6‐month (piece 2)										
Delta partner A	—	—	3.03 (1.46)*	0.08	*p* = 0.49	—	—	−0.07 (1.25)	−0.002	*p* = 0.04
Delta partner B	—	—	1.47 (1.47)	0.04		—	—	4.13 (1.29)*	0.12	
Model 3: relational aggression										
Baseline to 6‐month (full)										
Delta partner A	0.03 (0.03)	0.04	−0.15 (0.08)*	−0.07	*p* = 0.95	0.01 (0.03)	0.01	−0.08 (0.07)	−0.04	*p* = 0.27
Delta partner B	0.03 (0.03)	0.04	−0.15 (0.08)*	−0.07		0.07 (0.03)*	0.09	−0.20 (0.07)*	−0.11	
Actor effect slope *Χ* ^*2*^ diff^c^	*p* = 0.73									
Partner effect slope *Χ* ^*2*^ diff^d^	*p* = 0.53									
Baseline to 1‐month (piece 1)										
Delta partner A	—	—	−0.19 (0.08)*	−0.09	*p* = 0.82	—	—	−0.11 (0.08)	−0.05	*p* = 0.44
Delta partner B	—	—	−0.16 (0.09)*	−0.07		—	—	−0.20 (0.08)*	−0.10	
1‐ to 6‐month (piece 2)										
Delta partner A	—	—	−0.10 (0.11)	−0.04	*p* = 0.64	—	—	−0.06 (0.10)	−0.02	*p* = 0.26
Delta partner B	—	—	−0.17 (0.10)*	−0.07				−0.22 (0.08)*	−0.11	

*Note:* *Χ* ^*2*^ diff = Model test difference comparing actor and partner parameter estimates (*p* < 0.05 indicates parameters are *not* the same). The following *Χ* ^*2*^ diff superscripts indicate the parameters being compared: ^a^Delta Partner A ➔ Slope A vs. Delta Partner A ➔ Slope A; ^b^Delta Partner A ➔ Slope B vs. Delta Partner A ➔ Slope B; ^c^Delta Partner A ➔ Slope A vs. Delta Partner B ➔ Slope B; ^d^Delta Partner A ➔ Slope B vs. Delta Partner B ➔ Slope A. Constructive Communication (assessed by the Communication pattern questionnaire); Intimate Safety (assessed by the Intimacy Safety Questionnaire); Relational Aggression (assessed by the Conflict Tactic Scale); Delta A = T1 follow‐up assessment minus baseline assessment for participant A for the variable of interest for the given model; Delta B = T1 follow‐up assessment minus baseline assessment for participant B for the variable of interest for the given model. Control variables (couple poverty status, couple minority status, individual gender, and individual relationship distress) and correlation parameters are not reported in the table to ease interpretation. Effect sizes (i.e., Cohen's *d*) were used to compare partial model results and full model results. Cohen's *d* is a measure of effect size whereby standard cut‐offs for interpretation are small effect = 0.20, medium effect = 0.50, and large effect = 0.80.

* *p* < 0.05.

** *p* < 0.001.

We assessed study missingness at the individual level at both the 1‐month follow‐up assessment (34.7% missing) for all variables and the 6‐month follow‐up assessment (40.2% missing) for the primary outcome variable, relationship satisfaction. Most of our participants completed the intervention (89.9%). However, fewer persisted through the 1‐ and 6‐month feedback surveys. Missing data was handled within Mplus using full information maximum likelihood which assumes data missing at random. Therefore, control variables related to the pattern of missingness (i.e., couple poverty status, couple minority status, individual gender, and individual relationships distress) were included in each analysis.

## 3 Results

The first model, Model 1: Constructive Communication (see Table 2) indicated that improvement in partner A's constructive communication and partner B's constructive communication were both linked to improvements in partner A's relationship satisfaction over a 6‐month period. Results indicated both an actor and partner effect for partner A's relationship satisfaction, *β* = 0.30, *p* < 0.001; *β* = 0.28, *p* < 0.001, respectively. However, only improvement in partner B's constructive communication was linked to improvements in their own relationship satisfaction over a 6‐month period. Results indicated only an actor effect for partner B's relationship satisfaction, *β* = 0.43, *p* < 0.05. Delta scores were not linked to baseline relationship satisfaction for either partner A or partner B.

Comparison of the magnitude of change in piece 1 and piece 2 indicated that in the full constructive communication models (i.e., across all three times), the rate of change appears to decelerate from piece 1 (i.e., baseline to 1‐month) to piece 2 (i.e., 1‐ to 6‐month). However, partner B's change in constructive communication is linked to a similar rate of change for partner A's relationship satisfaction from piece 1 to piece 2. The *Χ* ^*2*^ difference tests indicate that, for CSI slope for partner A, actor and partner parameter estimates of constructive communication were significantly different for piece 1. However, for CSI slope for partner B, actor and partner parameters were significantly different for all models (full, piece 1, and piece 2). Additionally, *Χ* ^*2*^ difference tests indicate that the parameter estimate of Partner B's change in constructive communication linked to Partner A's relationship satisfaction is significantly different from the parameter estimate of Partner A's change in constructive communication linked to Partner B's satisfaction.

We observe a similar pattern in the next model, Model 2: Intimate Safety (see Table 2). Improvement in partner A's intimate safety and partner B's intimate safety were both linked to improvements in partner A's relationship satisfaction over a 6‐month period. Results indicated both an actor and partner effect for partner A's relationship satisfaction when considering a change in intimate safety, *β* = 0.39, *p* < 0.001; *β* = 0.30, *p* < 0.001, respectively. However, only improvement in partner B's intimate safety was linked to improvements in their own relationship satisfaction over a 6‐month period. Results indicated only an actor effect for partner B's relationship satisfaction, *β* = 0.45, *p* < 0.05. Additionally, improvements in partner B's emotional safety were linked to lower levels of their relationship satisfaction before the intervention, *β* = 0.30, *p* < 0.05.

Comparison of the magnitude of change in piece 1 and piece 2 indicated that in the full intimate safety models (i.e., across all three time points), the rate of change appears to decelerate from piece 1 (i.e., baseline to 1‐month) to piece 2 (i.e., 1‐ to 6‐month). The *Χ* ^*2*^ difference tests indicate that for CSI slope for partner A's actor and partner parameter estimates of intimate safety had no significant differences. However, for CSI slope for partner B actor and partner parameters were significantly different for all models (full, piece 1, and piece 2). Additionally, *Χ* ^*2*^ difference tests indicate that the parameter estimate of Partner B's change in intimate safety linked to Partner A's relationship satisfaction is significantly different from the parameter estimate of Partner A's change in intimate safety linked to Partner B's satisfaction.

For the final model, Model 3: Relational Aggression (see Table 2), we observed a similar pattern. Reductions in partner A's relational aggression and partner B's relational aggression were both linked to improvements in partner A's relationship satisfaction over a 6‐month period. Results indicated both an actor and partner effect for partner A's relationship satisfaction when considering a change in relationship aggression, *β* = −0.17, *p* < 0.05; *β* = −0.22, *p* < 0.05, respectively. However, only reductions in partner B's relational aggression were linked to improvements in their own relationship satisfaction over a 6‐month period. Results indicated only an actor effect with partner B's relationship satisfaction, *β* = −0.20, *p* < 0.05. Additionally, reductions in partner B's relational aggression were linked to higher levels of their own relationship satisfaction before the intervention, *β* = 0.30, *p* < 0.05.

Comparison of the magnitude of change in piece 1 and piece 2 indicated that in the full relational aggression models (i.e., across all three time points), there appears to be a similar rate of change in piece 1 and piece 2, which is a different pattern of change compared to constructive communication and intimacy safety. The exception to this is partner A's change in relational aggression, which is linked to a similar rate of change for their own relationship satisfaction from piece 1 to piece 2. The *Χ* ^*2*^ difference tests indicate that for CSI slope for partner A and partner B actor and partner relational aggression parameter estimates had no significant differences for any models (full, piece 1, and piece 2). Additionally, the *Χ* ^*2*^ difference tests indicate no significant differences when conducting comparisons across roles.

## 4 Discussion

The current study examined the associations between short‐term (baseline to 1‐month follow‐up) change in intimate safety, constructive communication, and relationship aggression and longer‐term (baseline to 6‐month follow‐up) change in relationship satisfaction for a community sample of couples who received the Relationship Checkup. Additionally, supplemental analyses were run with two partial models (baseline to 1‐month follow up, 1‐ to 6‐month follow up) to examine associations in change during the intervention (i.e., baseline to 1‐month follow up) and following the intervention (i.e., 1‐ to 6‐months follow up). The ability to examine these associations dyadically is a notable strength of this study. Specifically, we examine the association between short term change in relationship dynamics (i.e., constructive communication, intimate safety, relational aggression) and longer‐term change in relationship satisfaction for self and one's partner. All hypotheses were partially supported. For the partner who sought services (i.e., Partner A called to schedule the Relationship Checkup), improvement in indicators of relationship quality from baseline to 1‐month follow‐up was associated with improvements in relationship satisfaction for self and for one's partner from 1‐ to 6‐month follow‐up. However, for the other partner (i.e., Partner B did not seek out this intervention), improvement in relationship quality indicators from baseline to 1‐month follow‐up was associated with their own, but not their partner's, improvement in relationship satisfaction from 1‐ to 6‐month follow‐up. This might suggest differential effects of the intervention for the partner seeking out the Relationship Checkup on behalf of the relationship, and may be something important for clinicians delivering this intervention to pay attention to. For example, it may be that prioritizing the primary relationship concerns of the partner initiating the Relationship Checkup, while still fully attending to the concerns of the other partner, is a mechanism by which the Relationship Checkup is able to be both brief and effective. Thus, while it's important to attend to both partners in building rapport, paying close attention to the primary concerns for the partner who initiated services may be warranted. From a systemic perspective, addressing the most distressing concerns in the relationship will create positive change for both partners. Previous research with the Relationship Checkup found demographic differences (e.g., gender, race, income) predicted baseline help‐seeking attitudes for couple treatment (Wischkaemper et al. 2020). Taken together, these findings provide further support for clinicians to consider help‐seeking attitudes as an important factor influencing treatment outcomes. Future dyadic research is needed to more fully understand these patterns of change among partners and the factors that may be influencing these.

In a community sample with couples across the income spectrum, our findings suggest that improvements in intimate safety from baseline to 1‐month follow‐up may be linked to improvements in relationship satisfaction from 1‐ to 6‐month follow‐up. Although when comparing effect sizes, it appears there may be the potential for this improvement to slow down from 1‐ to 6‐month follow‐up. This finding makes sense when considering the context of which partner initiated the intervention. Specifically, for the partner who sought out the intervention (Partner A), improvements in intimate safety were associated with changes in relationship satisfaction directly after the intervention. However, this rate of change in satisfaction was not mirrored in the months following the intervention. It may suggest that for the partner initiating the intervention, their own satisfaction increases quickly during the intervention period and not as quickly after the intervention has concluded. We included several control variables to reduce the influence of other explanatory variables on our results, but we cannot definitively prove that these changes were caused by the Relationship Checkup itself. However, one prior study of the Relationship Checkup found that improvements in intimate safety marginally predicted improvements in relationship satisfaction (Hawrilenko et al. 2016), which provides more evidence that these changes may likely be due to the intervention. Furthermore, our results suggest that improvement in intimate safety from baseline to 6‐month follow‐up for Partner B (did not initiate the intervention) is linked to Partner A's (initiated the intervention) relationship satisfaction across this same timeframe. Future research is warranted to further understand the associations of change for the Relationship Checkup with regards to intimate safety and relationship satisfaction, with particular attention paid to how improvements in satisfaction differ between partners and are maintained over time.

Communication is a common target of change in couple interventions and is frequently examined as a mechanism of change for relationship satisfaction (Le et al. 2020). Prior research has produced mixed findings; some studies have found improvements in communication associated with improvements in satisfaction (e.g., Barton et al. 2017; Le et al. 2020), whereas others have not found an association among these variables at all (e.g., Williamson et al. 2016). The current study is the first known study with the Relationship Checkup to examine the association between short‐term changes in communication and longer‐term changes in relationship satisfaction. Findings suggest that improvements in communication from baseline to 1‐month follow‐up may be linked to improvements in relationship satisfaction from 1‐ to 6‐month follow‐up. Similarly to our findings for intimate safety, a comparison of effect sizes suggests there may be the potential for this improvement to slow down from 1‐ to 6‐month follow‐up. Again, this finding makes sense when considering the context of which partner initiated the intervention. Specifically, for the partner who sought out the intervention (Partner A), improvements in communication were associated with improvements in satisfaction directly following the intervention. However, this rate of change in satisfaction was not mirrored in the months following the intervention, which may suggest for the partner initiating the intervention their satisfaction increased quickly during the intervention period and not as quickly after the intervention concluded. Likewise, our results suggest that improvement in communication from baseline to 6‐month follow‐up for Partner B is linked to Partner A's relationship satisfaction. Thus, it may be particularly important to track improvements in relationship quality indicators (i.e., communication, intimate safety) for the partner who did not initiate the Relationship Checkup. Perhaps for the partner who sought out the Relationship Checkup, witnessing change in these indicators for their partner influences their own perception of satisfaction in the relationship. Further research is needed to more fully understand these associations over time, and the influence of help‐seeking (i.e., which partner schedules the Relationship Checkup) on this trajectory. A major strength of the Relationship Checkup is its ability to be tailored to address a couple's specific needs. Likewise, an inherent challenge of this is in measuring change when couples are receiving different types of intervention. For example, couples who indicated communication as a primary area of concern in their relationship may have received communication skills training as a part of their Relationship Checkup, while others did not. Future research should attempt to examine these change patterns within the context of the specific intervention(s) delivered within the Relationship Checkup.

Relationship aggression can be incredibly detrimental to individual (e.g., increased rates of depression and anxiety; Dillon et al. 2013) and relationship functioning (e.g., increased risk for relationship dissatisfaction and dissolution; Carroll et al. 2010). Research has demonstrated an inverse relationship between relationship aggression and relationship satisfaction such that higher rates of both psychological and physical aggression are associated with reports of lower relationship satisfaction (e.g., Curtis et al. 2016). Despite the established associations between these constructs, to the best of our knowledge this is the first study to examine the associations between short‐term change in relationship aggression and longer‐term change in relationship satisfaction in an assessment and feedback intervention, like the Relationship Checkup. In the current study, improvements in relationship aggression (i.e., decreased aggression) from baseline to 1‐month follow‐up were associated with improvements in relationship satisfaction from 1‐ to 6‐month follow‐up. Further, a comparison of effect sizes suggests there may be the potential for these improvements to be maintained from 1‐ to 6‐month follow‐up for the partner who did not seek out the intervention. Perhaps if the presence of relationship aggression contributed to the desire to seek out relationship help, these partners need more ongoing intervention, a greater attention to building up conflict resolution skills, or a “booster” session as the Relationship Checkup calls it, to maintain improvements over time. It is important to note here that couples in the current study were screened for intimate partner violence (IPV) at baseline and only couples not endorsing extreme emotional and/or physical safety concerns were retained. Thus, brief interventions like the Relationship Checkup may be appropriate for couples experiencing mild to moderate relationship aggression, and targeting reductions in aggression may be an avenue through which to enhance relationship satisfaction over time. Future research should aim to replicate these findings with couples across the income spectrum and with longer‐term follow‐up.

The results for each of the relationship variables examined in the current study are different, which indicates that these are independent constructs, but likely are interconnected over time. Previous research using latent profile analysis demonstrated that in some cases these relationship variables might group together while others do not (Roberson et al. 2020). Additionally, prior research supports the construct of intimate safety as distinct from relationship satisfaction (Hawrilenko et al. 2016). Thus, we believe there is enough evidence to suggest that these constructs are different but related, and future research should explore how changes in these relationship constructs are linked over time. This may be particularly important to identify which relationship construct(s) to target in interventions and if these constructs might differ between or within couples.

### 4.1 Limitations and Future Directions

Despite important contributions to the couple intervention literature, this study has some limitations. First, having communication, intimate safety, and relationship aggression data at just two points in time (baseline and 1‐month follow‐up) limits the degrees of freedom and thus our ability to more fully examine how changes in these variables are maintained and influence change in relationship satisfaction over time. Likewise, using delta scores to measure change in a variable between two points in time may not account for baseline differences or regression to the mean, potentially leading to biased estimates of true change. Future research should include at least three time points for all relationship quality variables over a longer period (e.g., at least 1 year) following the intervention. Second, while the current study extended prior research with the Relationship Checkup with couples across the income spectrum, our sample was limited in its inclusion of racial, ethnic, and sexual minoritized identities. Future studies across diverse groups are needed to more fully understand patterns of change for couples who receive the Relationship Checkup, and inform intervention adaptations for diverse populations. Additionally, while the funding mechanism for this study prohibited it, the lack of a control group is a notable limitation of the current study. Future research with the Relationship Checkup should consider including a control group to more clearly examine intervention effectiveness. However, it's also important to note that studies with waitlist controls show that individuals do not tend to spontaneously improve, and this is particularly true for more distressed individuals (e.g., Baucom et al. 2006). Finally, future research should examine additional mechanisms of change that cut across models of relationship intervention (e.g., understanding, emotion regulation, disclosure; Benson et al. 2012), and contribute to a more robust understanding of the ways in which the Relationship Checkup is an avenue for relationship improvement.

## 5 Conclusion

The current study examined the associations between short‐term changes in indicators of relationship quality (i.e., constructive communication, intimate safety, relationship aggression) and longer‐term change in relationship satisfaction with a community sample of couples who participated in the Relationship Checkup. This was the first known study to examine these associations with communication and relationship aggression for the Relationship Checkup. Additionally, this study sheds some light on the possible differing effects of being the initiator of this intervention on one's own and one's partner's trajectories. Findings suggest that short‐term improvements in communication, intimate safety, and aggression are associated with longer‐term improvements in relationship satisfaction. Specifically, improvements in these variables for self is associated with improvement in satisfaction for self and partner, only for the partner who sought out the Relationship Checkup. For the other partner, improvement in these variables is only associated with improvement in relationship satisfaction for self. Further, improvements in relationship aggression and satisfaction appear to be generally maintained over time, whereas improvements in intimate safety and constructive communication appear to generally decelerate following the intervention. Further research with the Relationship Checkup is needed to fully understand the associations in these improvements over time.

## Funding

Funding for this project was approved by the U.S. Department of Health and Human Services, Administration for Children and Families, Office of Family Assistance Grant #90FM0022 awarded to Kristina Coop Gordon.

## Conflicts of Interest

Co‐author James V. Cordova is co‐owner of a company (Arammu LLC) that trains practitioners to use the intervention used in the present study, the Checkup.

## Data Availability

All de‐identified data, code, and research materials are available upon request.
